# Probable Secondary Infections in Households of SARS Patients in Hong Kong

**DOI:** 10.3201/eid1002.030626

**Published:** 2004-02

**Authors:** Joseph T.F. Lau, Mason Lau, Jean H. Kim, Eric Wong, Hi-Yi Tsui, Thomas Tsang, Tze Wai Wong

**Affiliations:** * Chinese University of Hong Kong, Hong Kong Special Administrative Region, People’s Republic of China (SAR); † Department of Health, Hong Kong SAR; ‡ Chinese University of Hong Kong, Department of Community and Family Medicine, Hong Kong SAR

**Keywords:** SARS, secondary infections, epidemiology, Hong Kong

## Abstract

Although severe acute respiratory syndrome (SARS) is highly infectious in clinical settings, SARS has not been well examined in household settings. The household and household member attack rates were calculated for 1,214 SARS case-patients and their household members, stratified by two phases of the epidemic. A case-control analysis identified risk factors for secondary infection. Secondary infection occurred in 14.9% (22.1% versus 11% in earlier and later phases) of all households and 8% (11.7% versus 5.9% in the earlier and later phases) of all household members. Healthcare workers’ households were less likely to be affected. Risk factors from the multivariate analysis included at-home duration before hospitalization, hospital visitation to the SARS patient (and mask use during the visit), and frequency of close contact. SARS transmission at the household level was not negligible in Hong Kong. Transmission rates may be greatly reduced with precautionary measures taken by household members of SARS patients.

The first large-scale severe acute respiratory syndrome (SARS) outbreak occurred in the Prince of Wales Hospital in Hong Kong on approximately March 11, 2003 (*1*,*2*). It was followed by a large-scale community outbreak in the Amoy Gardens Estate, which had a total of 321 SARS cases as of April 15, 2003; 41.0% were in Block E residents (*3*). Environmental transmission of SARS was most likely primarily responsible for the Amoy Gardens outbreak (*4*,*5*). As of May 31, 2003, a total of 1,739 suspected or confirmed SARS cases were reported in Hong Kong, of which 384 were in hospital workers (22.1%) and approximately 321 were in residents of the Amoy Gardens (*6*) (Figure).

**Figure:**
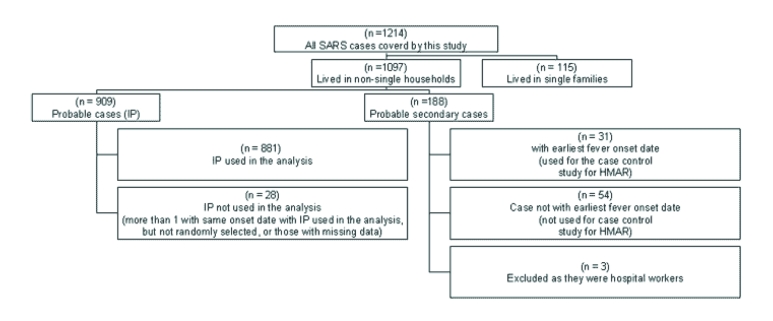
Distribution of the SARS patients covered in this study.

In the clinical setting, a very high attack rate of the SARS virus has been observed (*7*,*8*). However, few data describe the attack rates in community settings. The first objective of the study is to estimate the household attack rates and the household member attack rates for different categories of SARS patients. The second objective is to investigate risk factors associated with these two attack rates.

## Methods

### Study Population

The study population comprised all SARS case-patients who were reported to the Department of Health on or before May 16, 2003 (n = 1,690), and their household members (including kin, nonkin, and domestic helpers). In Hong Kong, confirmed or suspected SARS patients were defined as those with radiographic evidence of infiltrates consistent with pneumonia, and fever >38°C any time in the preceding 2 days, and at least two of the following symptoms: 1) history of chills in the past 2 days, 2) cough or breathing difficulty, 3) general malaise or myalgia, or 4) known history of exposure (*9*). This definition is the same as that of the World Health Organization for probable cases (*8*).

In this study, an index patient is defined as the SARS case-patient who had the earliest date of fever onset within a household. Household members who had an onset of symptoms later than the index patient are considered to be probable secondary (or tertiary) cases. Three of these cases were hospital workers who may have contracted SARS in the hospital setting and were hence excluded from the analysis.

### Data Collection

The list of telephone numbers, as well as some demographic and clinical background information of all probable SARS cases in Hong Kong (identified on or before May 16, 2003 [n = 1,690]), were obtained from the Department of Health. A team of trained interviewers called these numbers and briefed the person answering the telephone about the nature of the study. The interviewer then identified the person who had the earliest date of fever onset and confirmed that the household members had not been interviewed twice. When a household had two or more SARS cases with the same fever onset date (11 households), one of them was randomly selected as the index patient. Respondents were then requested to hand the telephone to the household member (who may or may not be the index patient) who was most familiar with the household situation to serve as the responder. The interview occurred at least 14 days after the index patient’s onset of symptoms past the maximum incubation period of 10 days.

By using a SARS registry, a research staff member later crosschecked that the index patient named by the interviewee was, in fact, the one with the earliest onset of fever, if there were more than one SARS case-patient in the household. In July, the names of all family members provided by the respondents were also checked against the registry to ensure that the study had not missed any probable secondary cases. This check also ensured that no duplicate interviews had been conducted.

The study was conducted from April 4, 2003, to June 10, 2003. Of the 1,690 probable SARS cases reported in Hong Kong as of May 16, a total of 1,214 (72%) SARS cases had been covered by the study (Figure). The 1,214 SARS cases came from 996 households (881 households were analyzed and 115 single households were excluded from the analysis). Of the remaining 476 reported SARS cases in Hong Kong that were not covered by this study, 140 case households (8.2%) did not have a correct telephone number, 163 (9.6%) could not be contacted after at least 5 different attempts, 163 (9.6%) refused to participate in the study, and 10 (0.6%) were not in Hong Kong or could not communicate in Chinese or English.

### Questionnaire

The study questionnaire collected the following information: 1) Sociodemographic data about the index patient and whether he or she resides in the Amoy Gardens (and apartment block number), 2) Household information—including all household members’ names, ages, gender, and relationship with the index patient, and if they were hospital workers, 3) Information about any “probable secondary SARS infection” among household members, 4) Data regarding individual household members’ hospital visits to the index patient, and 5) Data regarding close contact between individual household members and the index patient (Table 1).

**Table 1:** Univariate association between various risk factors and Household Member Attack Rates (HMAR)

Risk factor	% attack rate		Odds ratio (95% CI)	Chi-square p value
	Case	Control		
	(n = 131)	(n = 2,139)		
Sex^a^				
Male	46.6	48.3	1.00	0.701
Female	53.4	51.7	1.07 (0.75 to 1.53)	
Age (y)^b^				
18–30	46.6	46.9	1.00	0.287
31–40	15.3	15.3	1.17 (0.68 to 2.01)	
41–50	16.2	16.3	1.04 (0.60 to 1.81)	
51–60	10.9	10.7	1.58 (0.90 to 2.76)	
>61	11.1	10.8	1.65 (0.95 to 2.86)	
Type of Index Person (IP)				
Hospital care workers	7.6	33.5	1.00	<0.001^c^
Amoy Gardens Block E residents	10.7	2.8	16.99 (7.23 to 39.90)	
Amoy Gardens other Block residents	15.3	10.6	6.31 (2.91 to 13.67)	
Other community members	66.4	53.2	5.48 (2.83 to 10.61)	
Date of IP’s fever onset^d^				
Before March 25	51.9	34.2	1.00	<0.001
On or after March 25	48.1	65.8	0.48 (0.34 to 0.69)	
Duration IP stayed home between fever onset and hospitalization (d)^e^				
<2	31.3	51.0	1.00	<0.001
3–5	32.1	30.3	1.72 (1.11 to 2.68)	
>6	36.6	18.8	3.18 (2.07 to 4.90)	
IP visited by a family member during hospitalization?				
No	73.3	87.9	1.00	<0.001
Yes	26.7	12.1	2.65 (1.76 to 3.98)	
Mask use during hospital visits by a household member^f^				
Not visited by any household member	75.0	88.6	1.00	<0.001^3^
Visited, both with mask on	6.3	4.0	1.87 (0.88 to 3.96)	
Visited, one with mask on	5.5	3.6	1.78 (0.80 to 3.96)	
Visited, both without mask on	13.3	3.8	4.16 (2.37 to 7.30)	
Whether caretaker of IP				
No	64.9	82.0	1.00	<0.001
Yes	35.1	18.0	2.47 (1.70 to 3.60)	
Whether shared room and bed with IP^g^				
Never	59.7	81.3	1.00	<0.001
Sharing room	8.9	7.3	1.66 (0.86 to 3.19)	
Sharing room and bed	31.5	11.4	3.74 (2.48 to 5.64)	
Frequency of dining together with IP^h^				
Never	37.0	60.2	1.00	<0.001
<5	21.8	18.7	1.90 (1.15 to 3.12)	
5–10	14.3	9.7	2.40 (1.35 to 4.29)	
>10	26.9	11.4	3.82 (2.38 to 6.15)	
Frequency of close contact with IP (within 1 m)^i^				
Never	22.5	48.4	1.00	<0.001
Seldom	15.0	14.7	2.19 (1.19 to 4.02)	
Occasionally	24.2	16.4	3.17 (1.85 to 5.42)	
Frequent	38.3	20.5	4.03 (2.47 to 6.56)	
Frequency coughed at by IP (within 1 meter) ^j^				
Never	77.6	90.3	1.00	<0.001^3^
Seldom	6.5	4.2	1.81 (0.81 to 4.03)	
Occasionally	10.3	2.8	4.29 (2.17 to 8.48)	
Frequent	5.6	2.6	2.47 (1.03 to 5.90)	

^a^Information on 31 controls missing. ^b^Information on 7 cases and 160 controls missing . ^c^Chi-square test exact p value. ^d^Information on 3 controls missing. ^e^Information on 6 controls missing. ^f^Information on 3 cases 18 controls missing. ^g^Information on 7 cases and 24 controls missing. ^h^Information on 12 cases and 51 controls missing. ^i^Information on 13 cases and 37 controls missing. ^j^Information on 24 cases and 98 controls missing.

### Study Design

The household attack rate was defined as the number of households with at least one probable secondary SARS case divided by the total number of index patient’s households. The household member attack rate was defined as the total number of all probable secondary or tertiary SARS case-patients of all relevant index patient’s households divided by the total number of household members (not including the index patient) of all relevant index patient’s households.

Two analyses were performed to identify risk factors associated with household attack rates and household member attack rates. Households that had at least one probable secondary infection were first compared with those households which had no probable secondary infections in a number of risk or protective factors. To control for any period effects, a dummy variable was created to represent the two time periods (before March 25, 2003, and on or after March 25, 2003). March 25 corresponds to the beginning of the Amoy Gardens outbreak; after that date, public awareness of SARS was greatly heightened (*10*). The average number of secondary cases from one SARS-infected person declined greatly from 2.7 in the initial part of the epidemic to 0.9 after March 25 (*11*). (These figures were derived from modeling methods [instead of a survey] and were not confined to household cases; hence, they are not comparable to the results obtained by this study).

The second analysis used a case-control design that compared individual family members who were probable secondary SARS case-patients with those who were not. To avoid ambiguities arising from distinguishing secondary and tertiary infections, only the “first” probable secondary cases were used as a case in this case-control analysis, if there were multiple SARS cases in the household. In addition, this analysis also examined the frequency of close contacts between the case or control and the index patient (e.g., dining together, sharing a bedroom).

### Statistical Analyses

The household attack rates and the household member attack rates were calculated separately for four groups of index patients (hospital workers, Amoy Block E residents, other block residents, and other community members), and 95% confidence intervals (CI) were also derived. Univariate odds ratios and p values from chi-square test were obtained. Stepwise multivariate logistic regression methods using candidate variables that were, at a minimum, marginally significant in the univariate analysis (p <0.10) were conducted to obtain factors independently associated with household attack rates and household member attack rates. Statistical Package for the Social Sciences (SPSS), Chicago, IL, Version 11 was used for all analyses.

## Results

### Background Characteristics of Index Patients

Of the respondents, 54.6% were female and 45.4% were male; most index patients were 18 to 50 years of age. Healthcare workers represented almost one-third of the index patients and approximately 16% were Amoy Gardens Estate residents. Two-thirds of the index patients had fever onset during the later phase of the epidemic (on or after March 25), and most reported hospitalization within 5 days of fever onset (80.6%) and no hospital visits by household members (77.4%) (Table 2).

**Table 2:** Background characteristics of the Index Patient (IP)

Characteristic	n	%
Sex		
Male	400	45.4
Female	481	54.6
Age (y)^a^		
<18	44	5.1
18–30	239	27.8
31–40	197	22.9
41–50	165	19.2
51–60	76	8.8
>61	138	16.1
Education level^b^		
No education	60	7.1
Primary	152	17.9
1-F3	123	14.5
F4-F5	208	24.5
F6-F7	44	5.2
University or above	263	31.0
Type of IP		
Healthcare worker	267	30.3
Amoy Gardens Block E residents	36	4.1
Amoy Gardens other Block residents	107	12.1
Other community member	471	53.5
Duration IP stayed home between fever onset and hospitalization (d)^c^		
<2	440	50.1
3–5	268	30.5
>6	171	19.5
IP visited by any household member during hospitalization		
No	682	77.4
Yes	199	22.6
Date of IP’s fever onset^d^		
Before March 25	299	34.0
On or after March 25	581	66.0

^a^22 missing persons. ^b^31 missing persons. ^c^2 missing persons. ^d^1 missing person.

### Household Attack Rates

The overall household attack rate, as defined, was 14.9% (95% CI=12.6% to 17.4%) for all the households of the 881 index patients studied. Excluding households related to the Amoy Gardens, the household attack rate was 13.9% (96/738). The household attack rate was much higher for households of those index patients whose onset of fever occurred before March 25, 2003, than for those with onset of fever occurred on or after that date (22.4% versus 11.0%, OR = 0.43, p = 0.001). The Amoy Block E households had the highest household attack rate (38.9%), followed by those living in the other blocks of the Amoy Gardens (19.6%) and households of the “other community member” group (18.3%). The households with index patients who were healthcare workers had the lowest household attack rate (3.8%). Moreover, the household attack rates were higher for the earlier onset group as compared to the later onset group for all the four strata (Table 3).

**Table 3:** Household attack rates (HAR) and household member attack rates (HMAR) for different categories of index patient

	% attack rate		Overall	Odds ratio (95% CI)^a^	chi-square p value
Type of index patient	Date IP’s fever onset				
	<March 25, 2003	>March 25, 2003			
HAR					
Hospital workers	n = 114	n = 153	n = 267		
	7.0 (3.1–13.4)	1.3 (0.2–4.6)	3.8 (1.8–6.8)	0.18 (0.02 to 0.91)^b^	0.021
Other community members	n=148	n = 322	n = 471		
	29.1 (21.9–37.1)	13.4 (9.8–17.6)	18.3 (14.9–22.1)	0.38 (0.23 to 0.62)	<0.001
Amoy Gardens Block E residents	n = 12	n = 24	n = 36		
	50.0 (21.1– 78.9)	33.3 (15.6–55.3)	38.9 (23.1–56.5)	0.50 (0.10, to 2.54)	0.441^c^
Amoy Gardens other Block residents	n = 25	n = 82	n = 107		
	40.0 (21.1–61.3)	13.4 (6.9–22.7)	19.6 (12.6–28.4)	0.23 (0.07, 0.72)	0.008^c^
All households of all IP	n = 299	n = 581	n = 881		
	22.4 (17.8–27.6)	11.0 (8.6–13.9)	14.9 (12.6–17.4)	0.43 (0.29, 0.63)	<0.001
HMAR					
Hospital workers	n = 349	n = 381	n = 730		
	3.4 (1.8–5.9)	0.5 (0.06–1.9)	1.9 (1.1–3.2)	0.15 (0.02, 0.67)^2^	0.004
Other community members	n = 392	n = 866	n = 1,261		
	15.8 (12.4–19.8)	7.2 (5.5–9.1)	9.8 (8.3–11.6)	0.41 (0.28, 0.61)	<0.001
Amoy Gardens residents (Block E)	n = 27	n = 51	n = 78		
	37.0 (19.4–57.6)	17.7 (8.4–30.9)	24.4 (15.4–35.4)	0.36 (0.11, 1.19)	0.058
Amoy Gardens residents (non-Block E)	n = 59	n = 196	n = 255		
	22.0 (12.3–34.7)	7.7 (4.4–12.3)	11.0 (7.4–15.5)	0.29 (0.12, 0.71)	0.002
All households of all IP	n = 827	n = 1,494	n = 2,324		
	11.7 (9.6–14.1)	5.9 (4.8–7.2)	8.0 (6.9–9.1)	0.47 (0.34, 0.64)	<0.001

^a^The reference group is before March 25. ^b^Exact 95% CI. ^c^Fisher exact test p value

### Household Member Attack Rates

Among all 2,139 household members of the 881 index patients, a total of 188 (8%, 95% CI 7.0% to 9.2%) were probable secondary cases. The household member attack rates for the hospital healthcare worker group, the other community group, the Amoy non-Block E group, and the Amoy Block E group were 1.9%, 9.8%, 11%, and 24.4%, respectively. Excluding households related to Amoy Gardens, the household member attack rate was 6.9% (138/1,991). Similar period effects were observed: the odds ratios for comparing the two fever onset groups (on or after versus before March 25, 2003) were 0.15 (hospital healthcare worker group p = 0.004), 0.41 (other community group, p < 0.001), and 0.29 (Amoy non-E group, p = 0.002). For Amoy Block E respondents, the figures for the earlier and later onset groups were 37.1% and 17.7%, respectively (p = 0.058) (Table 3). The median duration between the date of onset of the index patients’ symptoms and their “first” probable secondary case was 6.5, 7.0, 2.0, and 4.0 days for the healthcare worker, other community members, Amoy Block E, and Amoy non-Block E groups, respectively.

### Factors Associated with Household Attack Rates

While sex of the index patient was not a significant factor, older age of index patient (OR = 1.57–3.77), type of index patient (OR = 5.74–16.35), longer duration home stay between fever onset and hospitalization (OR = 1.76–3.91), whether any household members visited the index patient (OR = 2.03), date of onset fever of index patient (later versus earlier onset groups, OR = 0.43) were all univariately associated with household attack rates (Table 4). Disinfection of the living quarter after the index patient's onset of fever was, however, not a significant factor (p = 0.88). All of these univariately significant variables except age were significant in the multivariate stepwise logistic regression (Table 5).

**Table 4:** Univariate analysis of associations between risk factors and Household Attack Rates (HAR)

Risk factor	Any probable secondary case within the household (%)		Odds ratio (95% CI)	Chi-square p value^a^
	Yes	No		
Sex of index person (IP)				
Male (n = 400)	16.5	83.5	1.00	0.215
Female (n = 481)	13.5	86.5	0.79 (0.55 to 1.15)	
Age of IP (y)^a^				
<30 (n = 283)	7.4	92.6	1.00	<0.001
31–40 (n = 197)	11.2	88.8	1.57 (0.84 to 2.93)	
41–50 (n = 165)	19.4	80.6	3.00 (1.67 to 5.41)	
51–60 (n = 76)	23.7	76.3	3.87 (1.94 to 7.73)	
>61 (n = 138)	23.2	76.8	3.77 (2.08 to 6.83)	
Type of IP				
Hospital workers (n = 267)	3.7	96.3	1.00	<0.001
Amoy Gardens bock E residents (n = 36)	38.9	61.1	16.35 (6.51 to 41.08)	
Amoy Gardens other Block residents (n = 107)	19.6	80.4	6.28 (2.84 to 13.85)	
Other community members (n = 471)	18.3	81.7	5.74 (2.93 to 11.26)	
Date of IP’s fever onset ^b^				
Before March 25 (n = 299)	22.4	77.6	1.00	<0.001
On or after March 25 (n = 581)	11.0	89.0	0.43 (0.29 to 0.62)	
Duration IP stayed home between fever onset and hospitalization (d)^c^				
<2 (n = 440)	9.3	90.7	1.00	<0.001
3–5 (n = 268)	15.3	84.7	1.76 (1.11 to 2.79)	
>6 (n = 171)	28.7	71.3	3.91 (2.46 to 6.20)	
IP visited by any household member during hospitalization?				
No (n = 682)	12.6	87.4	1.00	0.001
Yes (n = 199)	22.6	77.4	2.03 (1.36 to 3.03)	
Disinfection of IP’s quarters?				
Yes	15.2	84.8	1.00	0.884
No	14.7	85.3	0.96 (0.66 to 1.40)	

^a^Excluded 22 missing persons. ^b^Excluded 1 missing person. ^c^Excluded 2 missing persons.

**Table 5:** Summary of stepwise multivariate logistic regression model predicting “probable secondary infection” within the household level^a^

Risk factor	Coefficient	SE	Odds ratio (95% CI)	p value
Type of Index Person (IP)				
Healthcare worker			1.00	
Amoy Gardens Block E residents	3.074	0.487	21.62 (8.33 to 56.10)	<0.001
Amoy Gardens other Block residents	1.901	0.425	6.69 (2.91 to 15.39)	<0.001
Other community member	1.705	0.354	5.50 (2.75 to 11.01)	<0.001
Date of IP’s fever onset				
Before March 25			1.00	
On or after March 25	–0.696	0.235	0.50 (0.32 to 0.79)	<0.001
Duration IP stayed home between fever onset and hospitalization (d)				
<2			1.00	
3–5	0.283	0.258	1.33 (0.80 to 2.20)	0.274
>6	1.045	0.265	2.84 (1.69 to 4.78)	<0.001
IP visited by any household member when hospitalized?				
No			1.00	
Yes	0.483	0.242	1.62 (1.01 to 2.60)	0.046

^a^Age was not significant in the multivariable analysis.

### Factors Associated with Household Member Attack Rates

Similar to the case of probable secondary infection at the household attack rate, type of index patient (OR = 5.48–16.99, Table 1), whether the individual family member had visited the index patient in the hospital (OR= 2.65), longer duration of index patient's home stay (OR = 1.72 and 3.18), and index patient's date of fever onset (later versus earlier onset date, OR = 0.48) were univariately significant factors distinguishing between the case group and the control group. Moreover, the risk for SARS transmission was greatly increased when both the individual household member and the index patient were not wearing a mask during the hospital visit, (OR = 4.16, Table 1). In the univariate analyses, variables associated with close contacts with the index patient, such as the following: whether the was the main caregiver of the index patient (OR = 2.47), whether the participant shared a room or a bed with the index patient (OR 1.66 and 3.74), frequency of dining together with the index patient (OR 1.90 and 3.82, respectively, for those having dined 5–10 times and >10 times during the period between onset of fever of index patient and his or her hospital admission) and frequency of being coughed on by the index patient within one m (OR = 1.81 and 2.47, respectively, for responses of occasionally and frequently), were also significantly associated with household member attack rates.

In the multivariate analyses, the type of index patient (hospital workers, other community workers, and the like) was associated with household member attack rates, and the directions were the same as in the univariate analyses (Table 6). Moreover, individual household members who had visited the index patient when neither the index patient nor the visitor had worn a mask were more likely to have contracted SARS, when compared to those who had not visited the index patient (OR = 3.12, Table 6). Those household members who had had occasional or frequent close contacts of <1 m with the index patient were more likely than other household members to be included in the case group (OR = 2.14 and 2.30, Table 6). The household members were also less likely to have the index patient’s onset of fever occurring on or after March 25 as compared to the control group (OR= 0.51).

**Table 6:** Summary of multivariate logistic regression model predicting probable secondary infection” at household members (N = 2,195)

Risk factor	Coefficient	S.E.	Odds ratio (95% CI)	p value
Type of Index Person (IP)				
Hospital care workers			1.00	
Amoy Gardens Block E residents	2.888	0.455	17.95 (7.35 to 43.83)	<0.001
Amoy Gardens other Block residents	1.661	0.419	5.26 (2.32 to 11.95)	<0.001
Other community members	1.387	0.352	4.01 (2.01 to 7.98)	<0.001
IP visited by a household member				
Not visited by any			1.00	
Both with mask	0.571	0.412	1.77 (0.79 to 3.97)	0.166
Either one with mask	0.483	0.429	1.62 (0.70 to 3.76)	0.260
Both without mask	1.139	0.326	3.12 (1.65 to 5.91)	<0.001
Frequency of close contact with IP (within 1 m)^a^				
Never			1.00	
Seldom	0.466	0.338	1.59 (0.82 to 3.09)	0.168
Occasionally	0.762	0.304	2.14 (1.18 to 3.89)	0.012
Frequently	0.834	0.288	2.30 (1.31 to 4.05)	0.004
Date of IP’s fever onset				
Before March 25			1.00	
On or after March 25	–0.681	0.220	0.51 (0.33 to 0.78)	0.002
Duration Index person stayed home between fever onset and hospitalization (d)				
≤2			1.00	
3-5	0.092	0.278	1.10 (0.64 to 1.89)	0.740
≥ 6	0.655	0.278	1.93 (1.12 to 3.32)	0.018

^a^Information on 13 cases and 37 controls missing.

## Discussion

Of approximately 72% of SARS cases in Hong Kong (as of May 16, 2003) that were covered by this investigation, approximately 15% of all index patients’ households and 8% of all members of these households had contracted SARS. These figures include those of the Amoy Gardens residents. It is believed that the Block E transmissions had primarily resulted from environmental contamination rather than secondary infection (*4*,*5*). Excluding the Amoy Gardens cases, the attack rates were 13.9% and 8%, respectively. The SARS attack rates in the households therefore were not negligible.

The names of the probable secondary cases provided by the respondents were compared to the master list of known probable cases. A recent study, conducted by the Chinese University of Hong Kong, noted that none of the 94 asymptomatic family members of the SARS case-patients tested positive for SARS in serologic tests (unpub. data). Any underestimation due to asymptomatic transmission therefore should be minimal.

As the quarantine policy was only initiated on March 31 for the Amoy Gardens residents (*12*), the median home stay was longer for earlier onset SARS cases (4 days) than the later ones (2 days). Both the household and the household member attack rates were much higher in the initial phase of the epidemic (before March 25) (*10*). Moreover, between the first large-scale outbreak, which occurred approximately March 12, 2003, and March 25, 2003, relatively little was known about the disease, and hence minimal preventive measures against secondary infections were being practiced by household members (*10*).

Both the household and the household member attack rates of hospital healthcare workers were much lower than those of other types of households, even after controlling for other variables that were significant in the multivariate models. As compared to other households, less frequent close contacts were made in the healthcare worker households. Only 14% of the household members in the healthcare worker household had made frequent close contact (<1 m) with the index patient, as compared to 25% in the other groups (p < 0.01). Similarly, the percentages of dining together for >10 times during the reference period were 30.2% and 47.9%, respectively, for the healthcare worker and non-healthcare worker households (p < 0.01). These findings suggest that with a greater awareness and proper preventive measures, secondary attacks of SARS among household members may be greatly reduced.

Our data support the government’s suggestion that environmental contamination was responsible for the large number of SARS infections in the Amoy Gardens Block E (*4*,*5*) but not in other Blocks of the Amoy Gardens. The attack rates for the Amoy Block E households were much higher than those for households of other Blocks (for later onset households, household attack rates: 36% versus 13.4%; household member attack rates: 20.8% versus 7.7%), whereas the rates of the Amoy non-Block E households were comparable to those of the “other community group” (for later onset households, household attack rates: 13.4% versus 13.1%; household member attack rates: 7.7% versus 7.2%). The observation that the median duration between the onset of symptoms in the index patient and the “first” probable secondary case of the Amoy Gardens cases were much shorter than those of the other groups also supports the environmental contamination theory that had been suggested to explain the Amoy Gardens Block E outbreak.

Our data indicate that hospital visitations to the index patient was another independent risk factor for contracting SARS, suggesting that hospital visitors may have played an important role in the SARS epidemic in Hong Kong. Among all household members who had visited an index patient in the hospital, 51 (16.5%) of 310 contracted SARS (20.3% and 8.2%, respectively, for the earlier and later onset groups). Moreover, our results demonstrated that the risk was increased when both the SARS patient and the visitor were not wearing a mask. Hence, stringent hospital visitation policies should be implemented and proper personal protection equipment should be required for all visitors of SARS patients.

As a longer exposure period increased the risk for secondary SARS infection among household members, clear public health messages encouraging people who develop influenza-like symptoms to seek rapid medical treatment and to use preventive measures should be disseminated. An effective surveillance system should also be able to substantially reduce the duration of home stay of the SARS patients.

The frequency of close contact is another important risk factor for household member attack rates. Together with the significant association with index patient’s home stay duration, these results suggest that viral load is important in determining whether a secondary infection occurs. The results are also highly consistent with droplet theory of transmission but do not lend much support for transmission by fomites, particularly since the household attack rate was not found to be significantly associated with thorough disinfection of the living quarters.

When the data were stratified by Amoy Block E households versus other households, household disinfection was significantly associated with the household member attack rates in the former but not in the latter group (Amoy Gardens: OR=1.11; p=0.56,exact test; other households: OR = 0.24, p = 0.019, exact test; test for homogeneity, p = 0.013). Similar results were also obtained for the association between the household attack rate in the two groups (OR = 1.12 and 0.4, respectively, for Amoy Block E households and other households), although the association in the Amoy Block E group was not of statistical significance, possibly due to the small sample size (36 such households in total). This finding again strongly supports the claim that environmental contamination occurred in Amoy Block E households and that many of the cases were not secondary infections. Moreover, it suggests that although household disinfection was not a protective factor in the prevention of secondary infection, its role in reducing the risk for environmental infection cannot be dismissed. It is speculated that probable benefits of disinfection for protecting secondary infection might have been overridden by the effects of frequent contacts with the index patient or hospital visits.

The study has a few limitations. First, there is no way to confirm that the probable secondary infection of household members actually came from the index patient. Nosocomial infections, rather than secondary infections, may also have occurred in some of the household members during hospital visits to the index patient, but it is not possible to distinguish the two scenarios. The possibility of household members contracting the SARS virus in the community outside the home was, however, very small. Nevertheless, infection by environmental contamination has not been implicated as a large source of SARS except among Amoy Block E residents. Second, 44.6% of the time, information was provided by the household member most familiar with the household situation rather than the index patient. The households interviewed by the index patients and the households interviewed by proxy did not, however, differ in the distribution of risk factors. Moreover, most Hong Kong residents live in small apartments of <60 m^2^, and many avoided going out during the SARS epidemic; the people were very sensitized to close contact to those with SARS or flu-like symptoms (*10*). Hence, although the results may still be influenced by recall and reporting bias, the amount of bias should not substantially alter the findings. Third, even though recall bias may be another potential problem, almost all of the interviews were made within 3 weeks after the index patient’s onset of fever; given the extremely unusual nature of SARS, respondents should have been able to reliably recall the requested information. Fourth, the study was not able to cover all SARS patients in Hong Kong, but after incorrect or unavailable contact numbers were eliminated, 78.3% of all SARS patients had been covered by this study, and the refusal rate was moderate (10.5%). Finally, the case definition of SARS was nonspecific. Data on laboratory confirmation of the SARS coronavirus were not available so it was possible that some of the cases were in fact pneumonia rather than SARS. In the later phase of the epidemic, it was possible that either case-finding became more thorough or case-finding was more specific as more information became more available. Nevertheless, it is logical to argue that the secondary attack rate declined in the later phase as the awareness was greatly heightened. It is emphasized that the figures reported in this study are probable, rather than actual attack rates.

The study, being a large-scale study investigating SARS transmission in the community setting, allows us to have a better understanding of the infectivity, modes of transmission, and prevention of SARS in a community setting. It also gives insight into the prevention of secondary SARS infection within the household.
